# Effect of Argon as Filling Gas of the Storage Atmosphere on the Shelf-Life of Sourdough Bread—Case Study on PDO Tuscan Bread

**DOI:** 10.3390/foods11213470

**Published:** 2022-11-01

**Authors:** Alessandro Bianchi, Isabella Taglieri, Angela Zinnai, Monica Macaluso, Chiara Sanmartin, Francesca Venturi

**Affiliations:** 1 Department of Agriculture, Food and Environment, University of Pisa, Via del Borghetto 80, 56124 Pisa, Italy; 2 Interdepartmental Research Centre “Nutraceuticals and Food for Health”, University of Pisa, Via del Borghetto 80, 56124 Pisa, Italy

**Keywords:** modified atmosphere packaging, shelf-life, sourdough bread, staling, PDO Tuscan bread

## Abstract

The short shelf-life of PDO Tuscan bread limits its distribution to markets close to the production area, affecting its commercial success and the economic return by supply chain operators. While the application of MAP to store bread is widely accepted, the suitability of this technique to extend the shelf life of the PDO Tuscan bread is still to be explored. Furthermore, to the best of our knowledge no data are available in the literature about the use of argon as filling gas neither in pure atmosphere nor in combination with CO_2_. In this context, the aim of this study was to evaluate the effect of different modified packaging atmospheres on the shelf-life of sourdough bread. Slices of bread were stored individually in plastic bags at 23 °C in five different atmospheres (Ar (100%), N_2_ (100%), CO_2_ (100%), Mix CO_2_/N_2_ (70% CO_2_, 30% N_2_), Mix CO_2_/Ar (70% CO_2_, 30% Ar)), and Air was selected as a control. To select the best storage conditions, both chemical-physical, rheological, and organoleptic features were evaluated. Results showed that pure gases (CO_2_, N_2_, Ar) displayed good qualities as storage atmospheres compared to Air. In contrast, both Mix CO_2_/N_2_ and Mix CO_2_/Ar were the best in slowing down the staling process, thus doubling the shelf-life of bread, compared to other atmospheres. In conclusion, argon, as a preservation atmosphere, seems to be the best solution to extend the shelf-life of PDO Tuscan bread.

## 1. Introduction

Over the last decades, European consumer behavior in the baked goods category has changed and an increasing demand for products with reduced environmental impact together with symbolic features with ethical values such as naturalness, healthiness, distinctiveness, and consistency has been observed [1]. These new consumer concerns, together with the instability of the European/Italian wheat market, has improved the likelihood of success of rural systems and production strategies associated with traditional local productions with a cultural identity (i.e., traditional food products, geographical indications, etc.) [2].

Across Italy, in the last years, a re-localization process based on the establishment of Protected Designations of Origin (PDO) and Protected Geographical Indications (PGI) for bread was aimed at closing the gap between producers, processors, and consumers. The actuation of innovative, localized bread supply chains based on different assumptions such as variety, production method, baking process, zero-miles consumption models, etc. represents a possible strategy to sustain the cereal sector giving an economically viable alternative for Italian smallholders to compete on the market [1,3].

As in many other Italian regions, in Tuscany, bread has evolved as a basic consumption item in relation to the local history and culture. In order to foster and protect Tuscan bread, the process for the recognition of the “PDO Tuscan Bread” was promoted from 2002 by baker and farmer associations, the milling industry, and other local stakeholders [3]. The original recipe and the related product specification have been codified, starting from the specific varieties of wheat traditionally cultivated in Tuscany and to be used for the milling of wheat.

Some crucial aspects differentiate PDO Tuscan bread supply chains from conventional ones: (a) wheat must belong to a set of soft wheat varieties cultivated in Tuscany; (b) sourdough leavening is compulsory; (c) no salt can be added to the recipe; (d) flour must include wheat germ; (e) the final weight must range between 0.45 and 1.10 kg; and (f) the Consortium label is compulsory.

Thanks to the use of sourdough as leavening agent [4], the PDO Tuscan bread shows a significantly enhanced flavor complexity and higher savories than a widespread industrial white bread [5,6], even without any salt added in the formulation. Furthermore, the low pH together with high concentrations of lactic and acetic acid in the crumb can also explain the extended shelf life observed, mainly linked to the reduced mold spoilage as well as the slowing of the staling process [3,7,8,9,10].

Nevertheless, despite the improved shelf-life shown by PDO Tuscan bread, the geographic area of its supply chain appears still limited and a huge quantity of waste is generated with a consequent loss of economic resources together with a significant environmental impact [11,12].

As the refrigeration of freshly baked bread is not applicable as its texture and taste are negatively affected by low temperatures [13], the use of a proper modified atmosphere packaging (MAP) appears the best choice to extend the shelf life of the PDO Tuscan bread in order to maintain both sensorial features and nutritional value without using any preservatives [14,15], the use of which is not allowed by the traditional recipe.

Generally, when MAP is utilized to store bakery products, the gas mixture consists of 60% or more carbon dioxide (CO_2_) with nitrogen (N_2_) acting as a filler gas [16,17]. Similar to high-water content foods, CO_2_ can dissolve in the water of the bakery products to form carbonic acid, leading to a lowering of the pH. As reported by Smith et al. 1986 [18], the mold growth could only be delayed up to 5 and 10 days, but not prevented by N_2_ and/or CO_2_. In particular, a necessary condition to prevent mold growth was to maintain the level of O_2_ below 0.4%, in accordance with the results showing the reliance between O_2_ content and fungi growth on other types of products under MAP [19,20,21,22]. Nevertheless, the high concentration of CO_2_ may determine an increase in the perceived acidity to the taste [19,23,24,25,26]. Furthermore, the conclusive impact of CO_2_ in the MAP on the bread quality appears still contradictory [13].

In this context, the aim of this study was to evaluate the effect of different MAP on the shelf-life of the PDO Tuscan bread. Slices of bread were stored individually in plastic bags at 23 °C in five different atmospheres (Ar (100%), N_2_ (100%), CO_2_ (100%), Mix CO_2_/N_2_ (70% CO_2_; 30% N_2_), Mix CO_2_/Ar (70% CO_2_; 30% Ar)) and Air was utilized as control. To the best of our knowledge, no data are yet available in the literature concerning the feasibility to use Argon (Ar) as filling gas in MAP for sourdough bread storage. To select the best storage conditions, chemical-physical, rheological, and organoleptic features were evaluated.

## 2. Materials and Methods

### 2.1. Raw Materials

The flour (type 0) was obtained by a mix of four varieties (Bolero, Bologna, Verna, and Pandas) of common wheat (*Triticum aestivum*) produced by the Department of Agriculture, Food, Environment, and Forestry of the University of Florence during the 2021 crop season. The milling process was carried out at the Department of Agricultural, Food, and Environment (DAFE) of the University of Pisa using a commercial mill (Industry-Combi, Waldner Biotech, Lienz, Austria).

The chemical composition and the technological features of flour (Table 1) were determined according to the methods accepted by the International Organization for Standardization (ISO): humidity [27]; ashes [28]; proteins [29]; total fats [30]; falling number [31]; wet gluten and gluten index [32]; dry gluten [33]; total dietary fiber and sugars (sucrose, glucose, fructose, maltose) [34]; amylose and amylopectin [35]; total starch [36]; total polyphenols [37]; Chopin alveogram (W, P/L, P, L, G) [38]; Brabender farinogram (water absorption corrected to 14% humidity, dough time, stability, softening degree (E10: degree of softening after 10 min; E(ICC): softening degree 12 min, after max); and FQN: number of farinographic quality) [39].

### 2.2. Breadmaking Process

The sourdough utilized during the study was obtained by the Consortium for the protection of PDO Tuscan bread. The maintenance of sourdough was performed through consecutive back slopping in order to preserve the sourdough’s acidifying and leavening performances. Starter dough maintenance, back slopping, and baking were carried out under controlled operating conditions (time and temperature); bread making was carried out from a pre-ferment leavening agent, according to the two-step method of “biga” [8], as reported in Figure S1.

All the tests were conducted at the Food Technology laboratory of the Department of Agriculture Food and Environment of Pisa University; moreover, for each formulation, three replications were performed.

### 2.3. Bread Packaging and Storage

After baking, bread loaves were cooled for 2 h at room temperature, then sliced with an automatic slicing machine to 20 mm thickness. Each slice was packed individually in plastic bags (two plastic layers, outer nylon layer, Food Saver, Moncalieri, Torino, Italy), by an industrial packing machine (Lavezzini 450 GAS, Fiorenzuola d’Arda, Piacenza, Italy). During packaging, the composition of the internal atmosphere was modified to obtain 5 different MAP conditions (Ar (100%); N_2_ (100%), CO_2_ (100%), Mix CO_2_/N_2_ (70% CO_2_; 30% N_2_), Mix CO_2_/Ar (70% CO_2_; 30% Ar)); storage atmosphere composed by 100% Air was utilized as control.

Each pack was stored at a controlled temperature (T = 23 °C) during the whole observation period.

### 2.4. Bread Shelf-Life Assessment

To determine the bread shelf-life as a function of storage conditions, a total of 50 loaves (1 kg each) were prepared, 1500 slices were packed separately as described above, and divided into 6 groups as a function of MAP composition (5 different MAPs and Air 100% as control). To follow both the chemical-physical and sensory evolution of the packed bread during storage, as well as visible mold appearance on bread surface, four sliced samples for each storage condition were opened daily and analyzed as described below.

#### 2.4.1. Control of the Gaseous Atmosphere Inside the Packages

A Dansensor^®^ CheckPoint 3 CO_2_ (infrared sensor) and O_2_ (electrochemical sensor) (Ametek Mocon, Brooklyn Park, MN, USA) was used to measure the gas composition inside each pack during storage. The handheld non-destructive gas analyzer is easy to use, fast at processing data, and uses an optical sensor to provide the highest level of accuracy among similar products.

#### 2.4.2. Chemical Characterization of Bread

The moisture content of bread crumbs, taken from the center of each slice, was determined on approximately 5 g sample drying at 105 °C until constant weight, while the pH value was measured according to the AACC (American Association of Cereal Chemists) standard method, as previously reported [8]. Total titratable acidity (TTA) was determined following Gélinas et al. 1995 [40] The concentration of the main fermentative metabolites was investigated using specific enzymatic kits (Megazyme Ltd., Wicklow, Ireland), as described in Taglieri et al. 2020 [8].

#### 2.4.3. Weight Loss, Crumb Softness, and Water Activity (a_w_)

For each experimental run, the slices were weighed daily to assess the weight loss associated to water evaporation from the slices during storage; the value was expressed as a percentage reduction compared to the starting value.

a_w_ was measured by a HygroPalm HP23-AW-A equipment (Rotronic AG, Bassersdorf, Switzerland). The results were expressed as a percentage reduction in water activity compared to the starting value [41].

To measure the softness of the crumb, its compressibility was determined by a penetrometer PNR-12 (Anton Paar, Rivoli (TO), Italy) as described by Al Omari et al. (2016) [42] with some modifications: each sample was compressed in five spots by a weight of 90 g for 10 s. The compression spots were identified by holes, on the four corners and in the center, on a cardboard template, placed on the surface of each sample. The compressibility was measured in mm of penetration (0.1 mm = 1 penetration unit) and results were expressed as a percentage reduction in softness compared to the initial value.

#### 2.4.4. Mold Appearance

All the samples were checked daily for the presence of mold; each experimental run was stopped when 5% of the samples showed mold spoilage.

#### 2.4.5. Sensory Analyses

The sensory profiles of the bread samples were evaluated by a panel of 8 trained judges (aged between 23 and 60 years). All the people involved were members of the “Committee of Experts” of the Department of Agriculture, Food, and Environment Sciences of the University of Pisa. The tasting was carried out according to the previously developed protocol [8,43]. Before the tasting sections, a consensus panel was carried out to set up a sensory card specific for the shelf-life assessment. A final sensory sheet, including quantitative (visual aspect, olfaction, texture, taste, acidity, evolutionary state) together with hedonic ones (global pleasantness, overall acceptability), was individuated by agreement among panelists. Therefore, the overall hedonic index of bread was calculated [8], starting from the mean of the hedonic indices converted on a scale from 0 to 10, according to the following equation:

Overall hedonic index = Mean [Hedonic indexes] × 1.11 (1)

The research obtained the approval of the ethical committee of the University of Pisa (Comitato Bioetico dell’Università di Pisa, protocol n. 0088081/2020).

### 2.5. Statistical Analysis

The data obtained were processed by statistical analysis and the significance of differences among means was determined by one-way ANOVA (CoStat, Version 6.451, CoHort Software, Pacific Grove, CA, USA).

Chemical evaluations were performed at least in triplicate and the data are reported as average values. Tukey’s HSD test at *p* ≤ 0.05 significance was used for the separation of the samples.

The trend of the parameters over time was elaborated with the JMP software package version 17 (SAS Institute, Cary, NC, USA).

The results of the sensory analysis were processed by the Big Sensory Soft 2.0 software (version 2018). Sensory data were analyzed by two-way ANOVA with panelists and samples taken as main factors [44].

## 3. Results and Discussion

### 3.1. Weight Loss, Softness of the Crumb, and Water Activity Trend

After baking, the moisture redistribution from the crumb to the crust and the water loss that causes the decrease of softness of the crumb represent two critical issues deeply affecting bread shelf-life [7].

**Figure 1:**
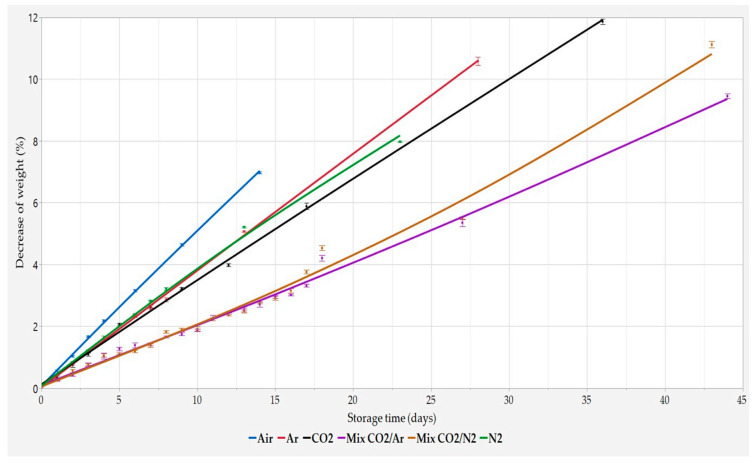
Percentage decrease in weight during storage time. Results are expressed as mean ± SD (n = 4).

**Figure 2:**
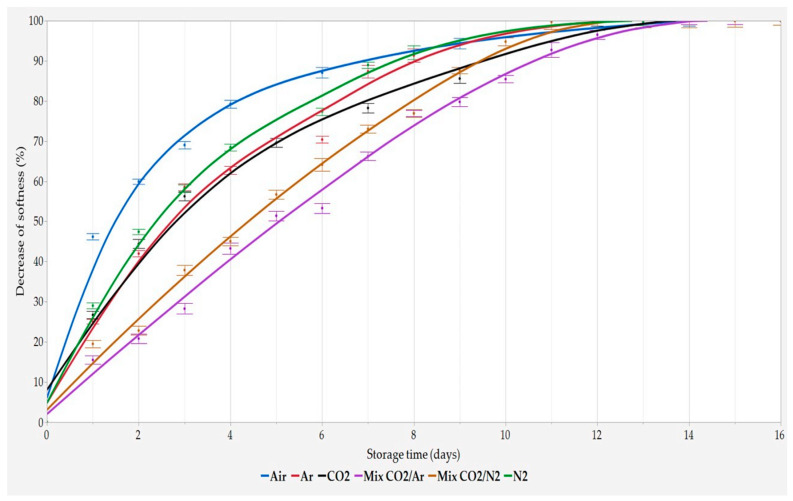
Percentage decrease of softness during storage time. Results are expressed as mean ± SD (n = 4).

As reported in Figure 1 and Figure 2, the decrease of both the weight loss and the softness of the crumb of the slices during storage showed the same trend as a function of MAP composition. When Air was used as storage atmosphere, the fastest decay rate was observed, while the lowest ones were observed when Mix CO_2_/N_2_ as well as Mix CO_2_/Ar were used, regardless of MAP composition.

Bread preserved with the three pure gas MAPs (Ar, N_2_, and CO_2_) exhibited an intermediate quality decay rate when compared to Air and both Mix, and very similar performances were observed regardless of the pure gas used for storage.

**Figure 3:**
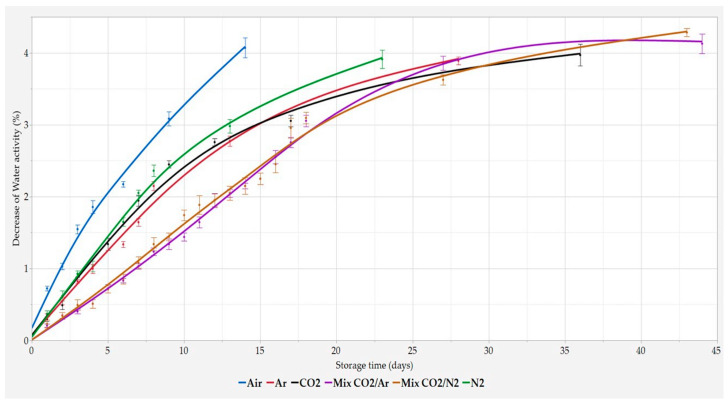
Percentage decrease in water activity during storage time. Results are expressed as mean ± SD (n = 4).

During storage, the free water moves inside the bread, with a flow going from the crumb to the crust. In the latter, the water tends to evaporate reducing the a_w_ with a variable intensity depending on the relative humidity present outside. In addition, the retrogradation of starch is one of the main issues of the staling process. Accordingly, a part of the water immobilized during the gelatinization of the starch becomes free during the recrystallization of the starch granules, thus increasing a_w_ [7,10,41]. Finally, in any time the a_w_ value of stored bread (Figure 3) gives a measure of the balance between these two opposite phenomena.

The a_w_ evolution followed the same trend previously reported for weight loss and crumb softness, with the best storage conditions obtained with both Mix CO_2_/N_2_ and Mix CO_2_/Ar, followed by the three pure gas MAPs, while Air was confirmed as the worst preserving atmosphere.

### 3.2. Mold Appearence

**Figure 4:**
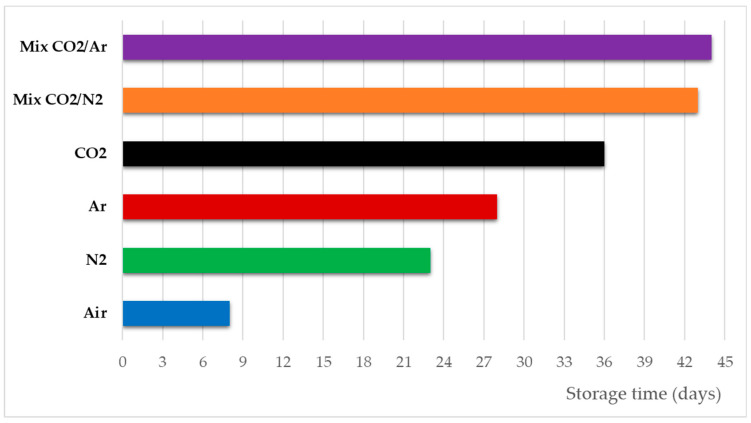
Period of time (days from packaging) during storage before the appearance of fungal bodies on 5% of bread samples, for each experimental condition.

As mold spoilage on the surface of bread deeply affects its organoleptic quality decay during storage, this parameter (Figure 4) can be used as marker of the acceptability limit as a function of the storage conditions.

As expected, mold spoilage was firstly evident when Air was used as storage atmosphere, thanks to the aerobic conditions provided in this experimental run.

Among the five different MAPs, the use of Mix CO_2_/Ar delayed the appearance of the molds, closely followed by Mix CO_2_/N_2_. Given the antimicrobial activity showed by CO_2_, MAP obtained by pure CO_2_ was the best one to counteract mold spoilage when compared with pure Ar and pure N_2_.

Furthermore, as the packaging utilized for storage did not allow water evaporation, the higher the water loss from the bread slice, the higher the relative humidity (RH) increasing inside the bag with consequent higher opportunity for mold development.

### 3.3. Sensory Evaluation

Panel tests were carried out on the slices of bread before packaging (t = 0) and daily, to evaluate the impact of different MAPs on the organoleptic profile of stored bread.

**Figure 5:**
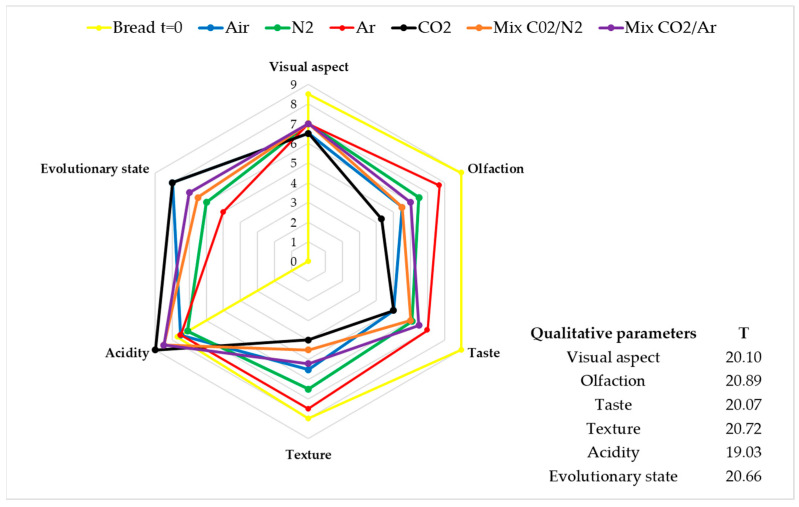
Median of significant qualitative parameters. Friedman’s ANOVA analysis (T > χ^2^, χ^2^ = 14.07).

Given that the experimental run with Air as preserving atmosphere was stopped at day 8 because of mold development, data related to the panel test made at day 8 for all the storage conditions were reported in Figure 5 and further discussed.

After 8 days of storage, the organoleptic profile of bread was significantly affected by the different MAPs, with the worst results observed when pure CO_2_ was utilized as preserving atmosphere, closely followed by Air. These results are consistent with what was previously observed [23,26]: the presence of CO_2_ in the storage atmosphere can significantly increase the sourness of the stored food with a negative impact on its taste. Furthermore, in all the other conditions (Air, Ar, N_2_), the value of the acid taste did not increase if compared to the value of bread at t = 0, regardless of the composition of filling atmosphere. In the case of sliced bread, the olfactory parameters were also affected, showing the highest decay. At last, the bread stored with pure Ar best retained its organoleptic profile when compared with bread at t = 0.

The hedonic quality level of a product is fundamental in determining its acceptability and overall pleasantness of the product. A value of 6 was taken as a reference point for the acceptability limit.

**Figure 6:**
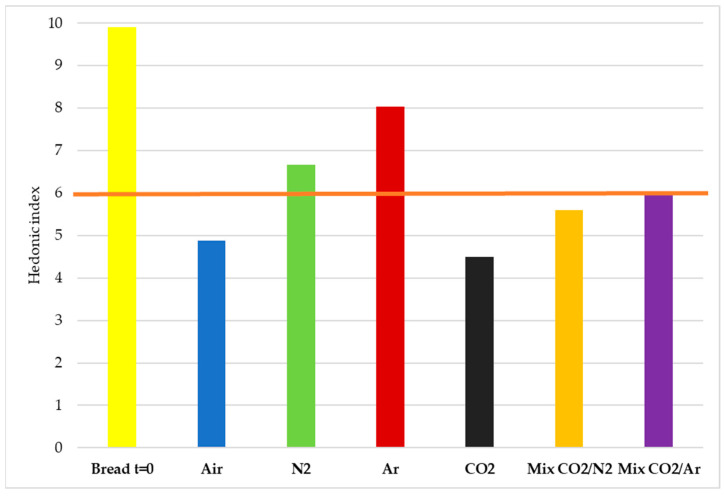
Hedonic index of the bread at time zero and after 8 days of storage in the different MAPs. The orange line indicates the acceptability limit value taken as a reference for this parameter.

The hedonic indexes of bread samples, at time zero and after 8 days of storage in the different experimental conditions, are reported in Figure 6.

The slices of bread preserved in Air and CO_2_ were clearly the least appreciated, while those preserved in pure argon were described as the most pleasant, closely followed by samples stored in pure N_2_. Furthermore, the presence of CO_2_ in the storage atmosphere, even in mix with N_2_ and Ar, reduced the pleasantness of the stored bread.

### 3.4. Chemical Characterization

Similar to what was reported for the sensory characterization of stored bread, the chemical composition of the bread samples was reported and discussed at time zero and after 8 days of storage under different MAPs (Table 2).

The time evolution of the chemical-physical parameters of the stored bread was significantly affected by the composition of the atmosphere inside the pack. In fact, as seen in the Table 2, pH, titratable acidity, and the other metabolites are statistically higher in the slices of bread stored in atmosphere containing CO_2_. Therefore, we can assume a dissolution of CO_2_ in the matrix, that, on a sourdough bread where the acidity is high results in a sum effect and determines an unacceptability from the sensory point of view. No significant differences were detected between N_2_ and Ar. Furthermore, after 8 days of storage, the evolution over time of the main chemical-physical parameters fully confirmed what was highlighted by the sensory analysis.

## 4. Conclusions

While the application of MAP to store bread is widely accepted, the suitability of this technique to extend the shelf life of PDO Tuscan bread is yet to be explored. Furthermore, to the best of our knowledge, no data are available in the literature on the use of argon as filling gas nor in pure atmosphere nor in mixture with other filling gas (i.e., CO_2_ or N_2_).

Based on the merging of the chemical-physical, rheological, and organoleptic data collected daily during this storage trial, it was possible to confirm that MAP is a proficiency strategy to significantly extend the shelf-life of this product, the worst results being observed in the control packaging (100% Air as filling gas).

According to the literature [19,23,24,25,26], in the storage conditions of this study, the presence of CO_2_ inside the package significantly affected the taste of the stored bread by increasing the perceived acidity. Given that the PDO Tuscan bread is a sourdough bread, the above-mentioned increase was even more evident due to the high acidity that characterized the bread before packaging.

All in all, the slowest quality decay rate was observed when 100% argon was utilized as filling gas. Thanks to the promising results obtained with pure argon, subsequent industrial-scale experiments will be carried out with different mixtures of Ar/N_2_, to identify also the best economic solution.

## Figures and Tables

**Table 1:** Chemical composition and technological features of flour. Results are expressed as mean ± SD (n = 4).

Parameters	Units	Flour
**Chemical**		
Humidity	% w/w	10.90 ± 0.30
Ashes	% w/w	1.37 ± 0.05
Proteins	% w/w	12.90 ± 0.31
Total fats	% w/w	2.53 ± 0.53
Total dietary fiber	% w/w	6.72 ± 0.22
Sucrose	% w/w	0.96 ± 0.05
Glucose	% w/w	0.43 ± 0.02
Fructose	% w/w	0.14 ± 0.01
Maltose	% w/w	6.28 ± 0.26
Wet gluten	% w/w	34.12 ± 2.02
Dry gluten	% w/w	10.94 ± 1.64
Gluten index	% w/w	75.32 ± 10.01
Amylose	% w/w	20.83 ± 0.23
Amylopectin	% w/w	79.23 ± 0.23
Total Starch	% w/w	83.72 ± 0.52
Total polyphenols	mg gallic acid/kg	833 ± 17
**Technological**		
W	10^−4^ joules	255 ± 29
P/L		3.3 ± 0.8
P	mm	152 ± 13
L	mm	48 ± 9
G		15.0 ± 1.6
Falling number	Seconds	327 ± 26
Water absorption	%	68.9 ± 0.7
Dough time	Minutes	4.3 ± 1.5
Stability	Minute	5.4 ± 2.7
E10	UF	58 ± 5
E(ICC)	UF	90 ± 23
FQN		75± 2

E10 = degree of softening after 10 min; E(ICC) = softening degree 12 min after max, FQN = number of farinographic quality; UF = Farinographic unit.

**Table 2:** Chemical composition of the sourdough bread produced at time zero and after 8 days of storage under different MAPs. Results are expressed as mean ± SD (n = 4).

Parameters	*p*-Value ^1^	t = 0	Air	CO_2_	N_2_	Ar	Mix CO_2_/Ar	Mix CO_2_/N_2_
% of dry matter (% dm)	***	61.63 ^d^	64.23 ^a^	63.48 ^bc^	63.62 ^b^	63.68 ^b^	63.34 ^c^	63.28 ^c^
pH	**	3.89 ^a^	3.81 ^ab^	3.76 ^c^	3.84 ^ab^	3.85 ^ab^	3.78 ^bc^	3.81 ^abc^
Total titratable acidity (meq lactic acid/g dm)	*	0.032 ^b^	0.034 ^ab^	0.036 ^a^	0.035 ^ab^	0.035 ^ab^	0.038 ^a^	0.036 ^a^
Acetic acid (mmol/g dm)	*	0.062 ^b^	0.076 ^ab^	0.086 ^a^	0.073 ^ab^	0.072 ^ab^	0.091 ^a^	0.093 ^a^
Lactic acid (mmol/g dm)	*	0.052 ^b^	0.057 ^ab^	0.060 ^a^	0.056 ^ab^	0.057 ^ab^	0.061 ^a^	0.059 ^a^
Ethanol (mmol/g dm)	n.s.	0.087	0.087	0.088	0.087	0.085	0.082	0.088

In the same row, different letters indicate significant difference among values. ^1^ Significance level: *** = *p* < 0.001; ** = *p* < 0.01; * = *p* < 0.05; n.s. = not significant (*p* > 0.05).

## Data Availability

Data is contained within the article.

## Supplementary Materials

The following supporting information can be downloaded at: https://www.mdpi.com/article/10.3390/foods11213470/s1, Figure S1: Baking protocol and operating conditions adopted for the experimental trials.

## References

[1] Sacchi G., Belletti G., Biancalani M., Lombardi G.V., Stefani G. (2019). The valorisation of wheat production through locally-based bread chains: Experiences from Tuscany. J. Rural Stud..

[2] Belletti G., Casabianca F., Marescotti A. (2012). Local food quality and local resources. Local Agri-Food Systems in a Global World: Market, Social and Environmental Challenges.

[3] Galli F., Venturi F., Bartolini F., Gava O., Zinnai A., Chiara S., Andrich G., Brunori G. (2017). Shaping food systems towards improved nutrition A case study on Tuscan Bread Protected Designation of Origin. Int. Food Agribus. Manag. Rev..

[4] Ma S., Wang Z., Guo X., Wang F., Huang J., Sun B., Wang X. (2021). Sourdough improves the quality of whole-wheat flour products: Mechanisms and challenges—A review. Food Chem..

[5] Malandrin V., Rossi A., Dvortsin L., Galli F., Csobán K., Könyves H.E., University of Debrecen, Litográfia Nyomda (2015). The Evolving Role of Bread in the Tuscan Gastronomic Culture. Gastronomy and Culture.

[6] Galli F., Brunori G. (2017). Sustainability Performance of Food Chains: Linking Biodiversity and Nutritional Value in Italian Wheat to Bread Chains. Adv. Food Secur. Sustain..

[7] Taglieri I., Macaluso M., Bianchi A., Sanmartin C., Quartacci M.F., Zinnai A., Venturi F. (2021). Overcoming bread quality decay concerns: Main issues for bread shelf life as a function of biological leavening agents and different extra ingredients used in formulation. A review. J. Sci. Food Agric..

[8] Taglieri I., Sanmartin C., Venturi F., Macaluso M., Zinnai A., Tavarini S., Serra A., Conte G., Flamini G., Angelini L.G. (2020). Effect of the leavening agent on the compositional and sensorial characteristics of bread fortified with flaxseed cake. Appl. Sci..

[9] Venturi F., Sanmartin C., Taglieri I., Nari A., Andrich G. (2016). Effect of the baking process on artisanal sourdough bread-making: A technological and sensory evaluation. Agrochimica.

[10] Rasmussen P.H., Hansen A. (2001). Staling of wheat bread stored in modified atmosphere. LWT Food Sci. Technol..

[11] Zingale S., Guarnaccia P., Matarazzo A., Lagioia G., Ingrao C. (2022). Science of the Total Environment A systematic literature review of life cycle assessments in the durum wheat sector. Sci. Total Environ..

[12] Brancoli P., Lundin M., Bolton K., Eriksson M. (2019). Bread loss rates at the supplier-retailer interface—Analysis of risk factors to support waste prevention measures. Resour. Conserv. Recycl..

[13] Upasen S., Wattanachai P. (2018). Packaging to prolong shelf life of preservative-free white bread. Heliyon.

[14] Kotsianis I.S., Giannou V., Tzia C. (2002). Production and packaging of bakery products using MAP technology. Trends Food Sci. Technol..

[15] Gutiérrez L., Sánchez C., Batlle R., Nerín C. (2009). New antimicrobial active package for bakery products. Trends Food Sci. Technol..

[16] Axel C., Zannini E., Arendt E.K. (2017). Mold spoilage of bread and its biopreservation: A review of current strategies for bread shelf life extension. Crit. Rev. Food Sci. Nutr..

[17] Fernandez U., Vodovotz Y., Courtney P., Pascall M.A. (2006). Extended shelf life of soy bread using modified atmosphere packaging. J. Food Prot..

[18] Smith J.P., Ooraikul B., Koersen W.J., Jackson E.D., Lawrence R.A. (1986). Novel approach to oxygen control in modified atmosphere packaging of bakery products. Food Microbiol..

[19] Stamatis N., Arkoudelos J. (2007). Quality assessment of Scomber colias japonicus under modified atmosphere and vacuum packaging. Food Control.

[20] El Halouat A., Debevere J.M. (1997). Effect of water activity, modified atmosphere packaging and storage temperature on spore germination of moulds isolated from prunes. Int. J. Food Microbiol..

[21] Sanguinetti A.M., Del Caro A., Scanu A., Fadda C., Milella G., Catzeddu P., Piga A. (2016). Extending the shelf life of gluten-free fresh filled pasta by modified atmosphere packaging. LWT Food Sci. Technol..

[22] Taniwaki M.H., Hocking A.D., Pitt J.I., Fleet G.H. (2001). Growth of fungi and mycotoxin production on cheese under modified atmospheres. Int. J. Food Microbiol..

[23] Sanmartin C., Venturi F., Macaluso M., Nari A., Quartacci M.F., Sgherri C., Flamini G., Taglieri I., Ascrizzi R., Andrich G. (2018). Preliminary Results About the Use of Argon and Carbon Dioxide in the Extra Virgin Olive Oil (EVOO) Storage to Extend Oil Shelf Life: Chemical and Sensorial Point of View. Eur. J. Lipid Sci. Technol..

[24] Suppakul P., Thanathammathorn T., Samerasut O., Khankaew S. (2016). Shelf life extension of “fios de ovos”, an intermediate-moisture egg-based dessert, by active and modified atmosphere packaging. Food Control.

[25] Fik M., Surówka K., Maciejaszek I., Macura M., Michalczyk M. (2012). Quality and shelf life of calcium-enriched wholemeal bread stored in a modified atmosphere. J. Cereal Sci..

[26] Zinnai A., Venturi F., Sanmartin C., Andrich G. (2012). Changes in physicochemical and sensory characteristics of fresh bread rolls maintained in different storage conditions. Agrochimica.

[27] (2009). Cereals and cereal products—Determination of moisture content.

[28] (2007). Cereals, Pulses and By-Products-Determination of Ash Yield by Incineration.

[29] (2013). Cereals and pulses—Determination of the Nitrogen Content and Calculation of the Crude Protein Content—Kjeldahl Method.

[30] (2015). Cereals, Cereals-Based Products and Animal Feeding Stuffs—Determination of Crude Fat and Total Fat Content by the Randall Extraction Method.

[31] (2009). Wheat, Rye and Their Flours, Durum Wheat and Durum Wheat Semolina—Determination of the Falling Number According to Hagberg-Perten.

[32] (2015). Wheat and Wheat Flour—Gluten Content—Part 2: Determination of Wet Gluten and Gluten Index by Mechanical Means.

[33] (2006). Wheat and Wheat Flour—Gluten Content—Part 3: Determination of Dry Gluten from Wet Gluten by an Oven Drying Method.

[34] Novotni D., Mutak N., Nanjara L., Drakula S., Čukelj Mustač N., Voučko B., Ćurić D. (2020). Sourdough fermentation of carob flour and its application in wheat bread. Food Technol. Biotechnol..

[35] Nivelle M.A., Beghin A.S., Vrinten P., Nakamura T., Delcour J.A. (2020). Amylose and amylopectin functionality during storage of bread prepared from flour of wheat containing unique starches. Food Chem..

[36] McCleary B.V., Solah V., Gibson T.S. (1994). Quantitative measurement of total starch in cereal flours and products. J. Cereal Sci..

[37] Li Y., Ma D., Sun D., Wang C., Zhang J., Xie Y., Guo T. (2015). Total phenolic, flavonoid content, and antioxidant activity of flour, noodles, and steamed bread made from different colored wheat grains by three milling methods. Crop J..

[38] (2015). Cereals and Cereal Products—Common Wheat (Triticum aestivum L.)—Determination of Alveograph Properties of Dough at Constant Hydration from Commercial or Test Flours and Test Milling Methodology.

[39] (2013). Wheat Flour—Physical Characteristics of Doughs—Part 1: Determination of Water Absorption and Rheological Properties Using a Farinograph.

[40] Gélinas P., Audet J., Lachance O., Vachon M. (1995). Fermented Dairy Ingredients for Bread: Effects on Dough Rheology and Bread Characteristics. Cereal Chem..

[41] Nyangena I., Owino W., Ambuko J., Imathiu S. (2019). Effect of selected pretreatments prior to drying on physical quality attributes of dried mango chips. J. Food Sci. Technol..

[42] Al Omari D.Z., Abdul-Hussain S.S., Ajo R.Y. (2016). Germinated lupin (Lupinus albus) flour improves Arabic flat bread properties. Qual. Assur. Saf. Crop. Foods.

[43] Taglieri I., Sanmartin C., Venturi F., Macaluso M., Bianchi A., Sgherri C., Quartacci M.F., De Leo M., Pistelli L., Palla F. (2021). Bread fortified with cooked purple potato flour and citrus albedo: An evaluation of its compositional and sensorial properties. Foods.

[44] Hasted A. (2018). Statistical Analysis of Descriptive Data. Descriptive Analysis in Sensory Evaluation.

