# Supplementary figures and images for: Effect of Argon as Filling Gas of the Storage Atmosphere on the Shelf-Life of Sourdough Bread—Case Study on PDO Tuscan Bread
Source: Foods. 2022 Nov 1;11(21):3470. doi: 10.3390/foods11213470 (PMC9654732; doi:10.3390/foods11213470)

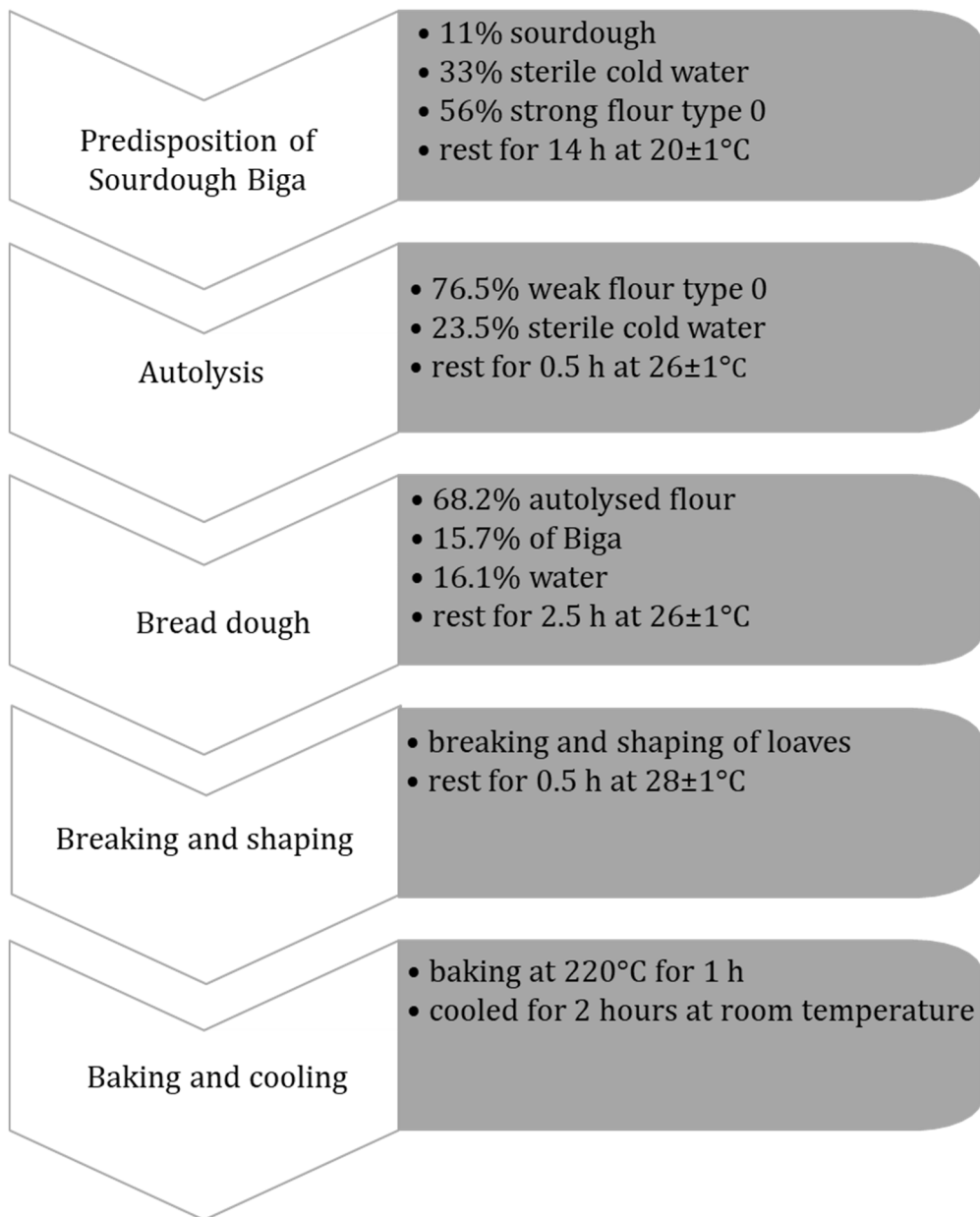

**Figure S1.** Baking protocol and operating conditions adopted for the experimental trials.

Supplement: Supplementary file 1 [file foods-11-03470-s001.zip › foods-1950966-supplementary.pdf]
